# Prevalence and Clinical Features of Uterine Isthmocele Following Cesarean Sections: An Observational Study at Rabia Balkhi Hospital in Afghanistan

**DOI:** 10.7759/cureus.74610

**Published:** 2024-11-27

**Authors:** Malalai Alami, Bibi Sarah Yousofzai, Rana Beloulou Latoui, Asma Abbas, Ruqiya Bibi, Muhammad Subhan, Seth Omari Mensah, Ernette Espiegle, Atinder Singh, Talieh Norouzi

**Affiliations:** 1 Obstetrics and Gynecology, Rabia Balkhi Hospital, Kabul, AFG; 2 Obstetrics and Gynecology, Be Team International Cure Hospital, Kabul, AFG; 3 Obstetrics and Gynecology, CHU Ibn Rochd Hospital, Annaba, DZA; 4 Obstetrics and Gynecology, Surraya Azeem Hospital Lahore, Lahore, PAK; 5 Medicine, Allama Iqbal Medical College, Lahore, PAK; 6 Obstetrics and Gynecology, Royal Free Hospital, London, GBR; 7 Internal Medicine, State University of Haiti, Port-au-Prince, HTI; 8 Medicine, World College of Medical Sciences and Research and Hospital, Gurugram, IND; 9 Family Medicine, Fasa Medical School, Mashhad, IRN

**Keywords:** cesarean section, diverticulum, isthmocele, metrorrhagia, placenta accrete

## Abstract

Background

Uterine isthmocele, a defect in the uterine wall at the cesarean scar, is increasingly recognized due to the rising rate of cesarean deliveries. Often asymptomatic, it may lead to complications such as abnormal bleeding, chronic pelvic pain, secondary infertility, or uterine rupture during subsequent pregnancies.

Objective

This study aimed to assess the prevalence, clinical features, and associated risk factors of uterine isthmocele among women with previous cesarean scars over four years (2019-2023) at Rabia Balkhi Hospital, Afghanistan.

Methods

This observational study analyzed 9,207 women with prior cesarean sections, including 4,540 cases retrospectively (2019-2021) and 4,667 cases prospectively (2021-2023). The diagnosis was based on clinical evaluations and transvaginal ultrasonography. Data were analyzed using MS Excel (Microsoft Corporation, Redmond, USA), with statistical significance assessed for symptom associations and risk factors.

Results

Uterine isthmocele was identified in 58 women (0.63%). The most common symptom was abnormal uterine bleeding (78%, p < 0.001), followed by dysmenorrhea (48%, p = 0.793), chronic pelvic pain (31%, p = 0.004), secondary infertility (22%, p < 0.001), and hypovolemic shock (8.6%, p < 0.001). Women with three cesarean scars had the highest prevalence of isthmocele (46.5%, p < 0.001). Ultrasonography was effective in detecting isthmocele and evaluating post-surgical outcomes.

Conclusion

The study highlights the need for improved cesarean techniques, early ultrasonographic diagnosis, and standardized management protocols for uterine isthmocele. These findings provide a foundation for optimizing care in women with multiple cesarean scars and underscore the importance of early intervention to prevent complications.

## Introduction

Uterine isthmocele, more commonly referred to as a pouch-like uterine niche, is a defect located on the inner wall near the cesarean scar area and generally measures between 1 and 2 millimeters deep at the isthmus border, first identified by Dr. Poidevin in 1961 [1]. This iatrogenic condition results from incomplete healing of cesarean scars. It may be caused by factors such as incomplete repair, reduced myometrial thickness, blood loss during cesarean surgery, additional hemostatic sutures to support blood loss control, endometritis, retroflexed uterus, or multiple scars [2]. With rising cesarean rates, isthmocele has increased at an alarming rate, impacting 19-88% of cases, with nearly 100% occurring after three cesareans [3]. Complications associated with surgery may include placenta accreta, pregnancy in the scar area, rupture of subsequent pregnancies, and secondary infertility [4]. Primary causes include incomplete repair of the hysterotomy, residual myometrial thickness, and reduced total myometrial thickness above the uterine scar [5]. Contributing factors may consist of suture type, single-layer uterine closure with locking sutures, low cesarean incision position, non-repair of the peritoneum, blood loss, additional hemostatic sutures needed, endometritis, anterior uterine wall adhesions, and genetic predisposition [6]. Risk factors for uterine rupture can include the position, multiple cesarean scars, and method of uterine closure - single-layer locked closure increasing rupture risk by four times compared to double layer [7]. Clinically, isthmocele can often remain undetected; however, symptoms may include dyspareunia, chronic pelvic pain, dysmenorrhea, abnormal uterine bleeding (AUB), dark red or brown discharge post-menstruation with subsequent spotting, and secondary infertility [8]. Diagnosing pelvic floor disorders requires an evaluation of history and symptoms as well as medical examinations such as transvaginal ultrasound (TVUS), saline infusion sonohysterography (SIS), sonohysterography (SHG), hysterosalpingography (HSG), magnetic resonance imaging (MRI), and diagnostic hysteroscopy, with TVUS providing a hypoechoic view of any defects [9,10]. Remedy for defects depends on their size, symptoms, and depth; options include medical management with oral contraceptives and levonorgestrel intrauterine devices, as well as surgical options like laparotomy, laparoscopy, vaginal repair, or hysteroscopic resection [10]. Untreated isthmocele can result in prolonged postmenstrual spotting, pelvic pain, and infertility - with more significant defects collecting more blood [11-12]. The current study seeks to examine the incidence, risk factors, clinical features, and management strategies for isthmocele cases at Rabia Balkhi Hospital, Afghanistan, from 2019 to 2023.

## Materials and methods

This study was approved by the Institutional Review Board (IRB) of the Ministry of Public Health of Afghanistan under approval number 2019/IRB/037. This four-year observational study was conducted at Rabia Balkhi Hospital from December 2019 to December 2023, aimed at identifying the prevalence and clinical features of uterine isthmocele among women with previous cesarean sections. The study used both retrospective (2019-2021) and prospective (2021-2023) approaches to examine patient records and conduct real-time evaluations, respectively.

Study design and population

Inclusion criteria targeted women aged 24 to 60 years with a history of at least one prior cesarean section and clinical signs suggestive of isthmocele, such as AUB, chronic pelvic pain, or secondary infertility. Exclusion criteria ruled out patients undergoing their first cesarean section, those without isthmocele-related symptoms, or those outside the specified age range or unwilling to participate.

Retrospective component (2019-2021)

In the retrospective phase, medical records of women who underwent cesarean sections between 2019 and 2021 were reviewed to identify cases of isthmocele. Data collected included patient demographics, cesarean history, postoperative complications, and any documented findings related to isthmocele. The mean age of patients in this group was 34.5 ± 6.2 years. TVUS evaluations were conducted on average 6-12 months after the cesarean section. This timeframe was based on available records, documenting evaluations within a year after the most recent cesarean.

Prospective component (2021-2023)

In the prospective phase, women with a history of cesarean section who presented to the hospital with symptoms suggestive of isthmocele were evaluated through clinical interviews, physical examinations, and targeted imaging assessments. TVUS was systematically performed within 6-12 months post-cesarean to assess scar integrity and identify any developing isthmocele. Real-time data, including ultrasound findings, clinical presentation, and patient-reported symptoms, were documented. The average age of patients in the prospective cohort was 33.7 ± 5.9 years.

Diagnostic procedures

TVS was used to diagnose isthmocele in both the retrospective and prospective phases, allowing precise visualization of the uterine scar area. No standardized grading criteria for isthmocele were applied in this study; instead, a descriptive approach was taken, documenting the presence and characteristics of each detected isthmocele.

Data collection and analysis

Data from the retrospective and prospective groups were collected and systematically analyzed using MS Excel. Statistical analysis was performed using chi-square and t-tests to compare incidences and clinical characteristics. The significance threshold was set at p < 0.05.

## Results

This four-year study analyzed the incidence and symptomatology of uterine isthmocele among women with prior cesarean sections at Rabia Balkhi Hospital. A total of 21,165 cesarean sections were performed between 2019 and 2023, with 9,207 (43.5%) involving patients with previous cesarean sections. Data were gathered retrospectively from hospital records (2019-2021) and prospectively through clinical evaluations (2021-2023), providing insights into demographic characteristics, incidence rates, and common symptoms associated with isthmocele.

Retrospective group (2019-2021)

In the retrospective analysis, patient records of those who had prior cesarean sections were reviewed. Demographic data, ultrasound findings, and basic clinical presentations were gathered without detailed follow-up on the type of cesarean section or postoperative complications. The average age of this group was 34.5 ± 6.2 years.

A total of 4,540 cases were evaluated, with 28 cases (0.62%) identified as isthmocele. Among this group, the most frequently reported symptoms were AUB (64%), dysmenorrhea (50%), and chronic pelvic pain (29%).

Prospective group (2021-2023)

The prospective phase involved active clinical evaluations through patient interviews, physical exams, and targeted ultrasound imaging. Myometrial thickness at cesarean scar sites was measured at an average interval of 6-12 months post-surgery to assess for potential isthmocele.

A total of 4,667 cases were evaluated, with 30 cases (0.64%) identified as isthmocele. The most commonly reported symptoms in this group included AUB (90%), dysmenorrhea (47%), chronic pelvic pain (33%), hypovolemic shock (10%), and secondary infertility (27%).

Incidence of isthmocele

**Figure 1:**
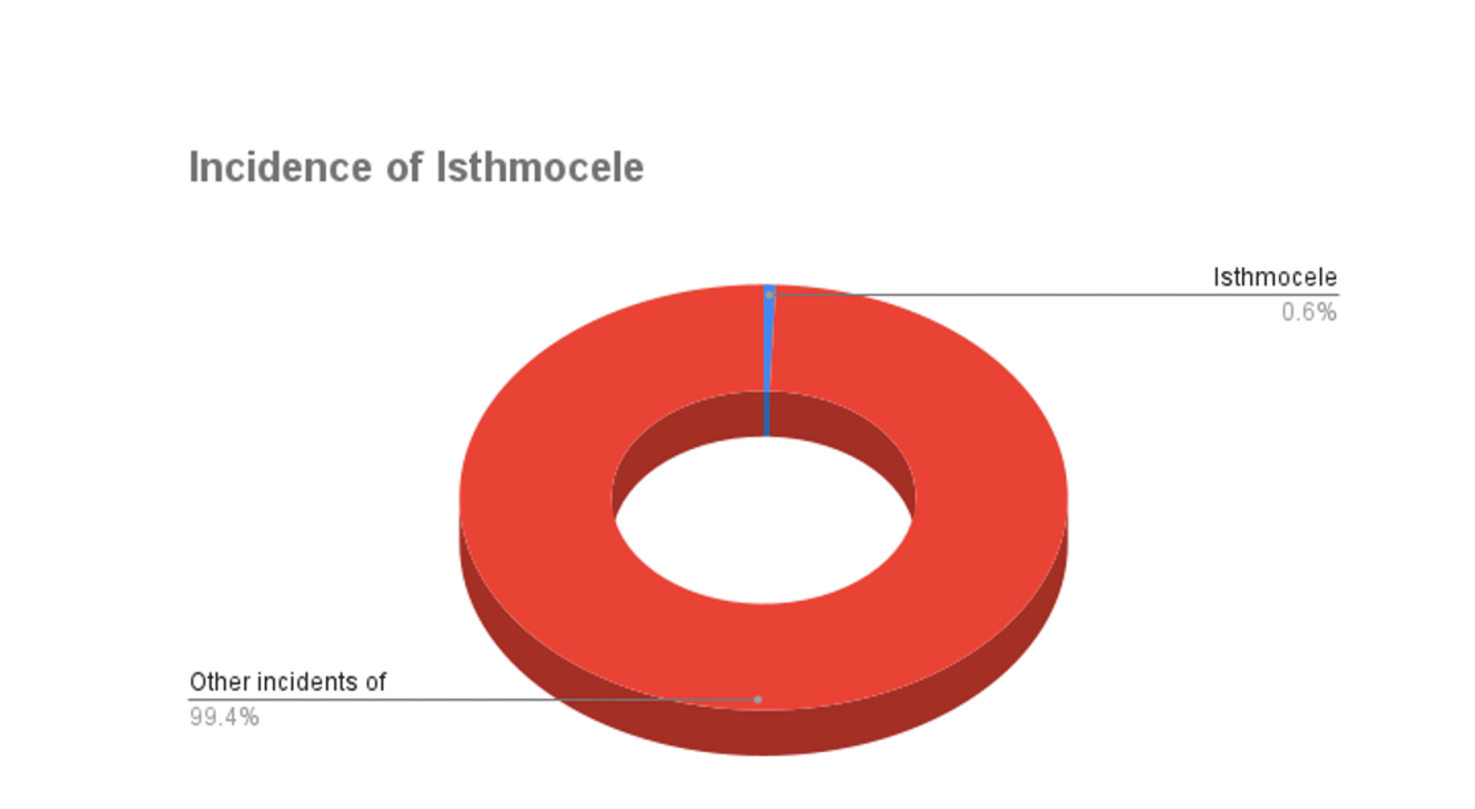
Incidence of isthmocele in our study

Among 21,165 cesarean sections performed during the study period, 9,207 (43.5%) involved patients with prior cesarean sections. A total of 58 cases of isthmocele were identified, yielding an overall prevalence of 0.62%. The incidence was comparable between the retrospective (0.62%) and prospective (0.64%) phases (p = 0.979). Table 1 and Figure 1 provide an overview of isthmocele cases within our study population versus other cesarean scar incidents.

**Table 1:** Incidence of isthmocele

Indicator	Number	Percentage (%)
Isthmocele	58	0.62
Other incidents of previous cesarean scars	9,149	99.38
Total number of cases of last cesarean scars	9,207	100

Table 2 shows the distribution of isthmocele cases by the number of cesarean scars, demonstrating a significant correlation (p = 0.0001). The mean number of scars in patients with isthmocele was 2.7 ± 0.9.

**Table 2:** Distribution of isthmocele cases by number of scars It indicates that individuals with three cesarean scars had the most significant number of complications, and three cases (5%) involved ectopic pregnancies at the site of one or more prior scars.

Number of cesarean scars	Cases	Percentage (%)
1	8	13.7
2	18	31
3	27	46.5
More than 3	5	8.6

Symptom prevalence

**Figure 2:**
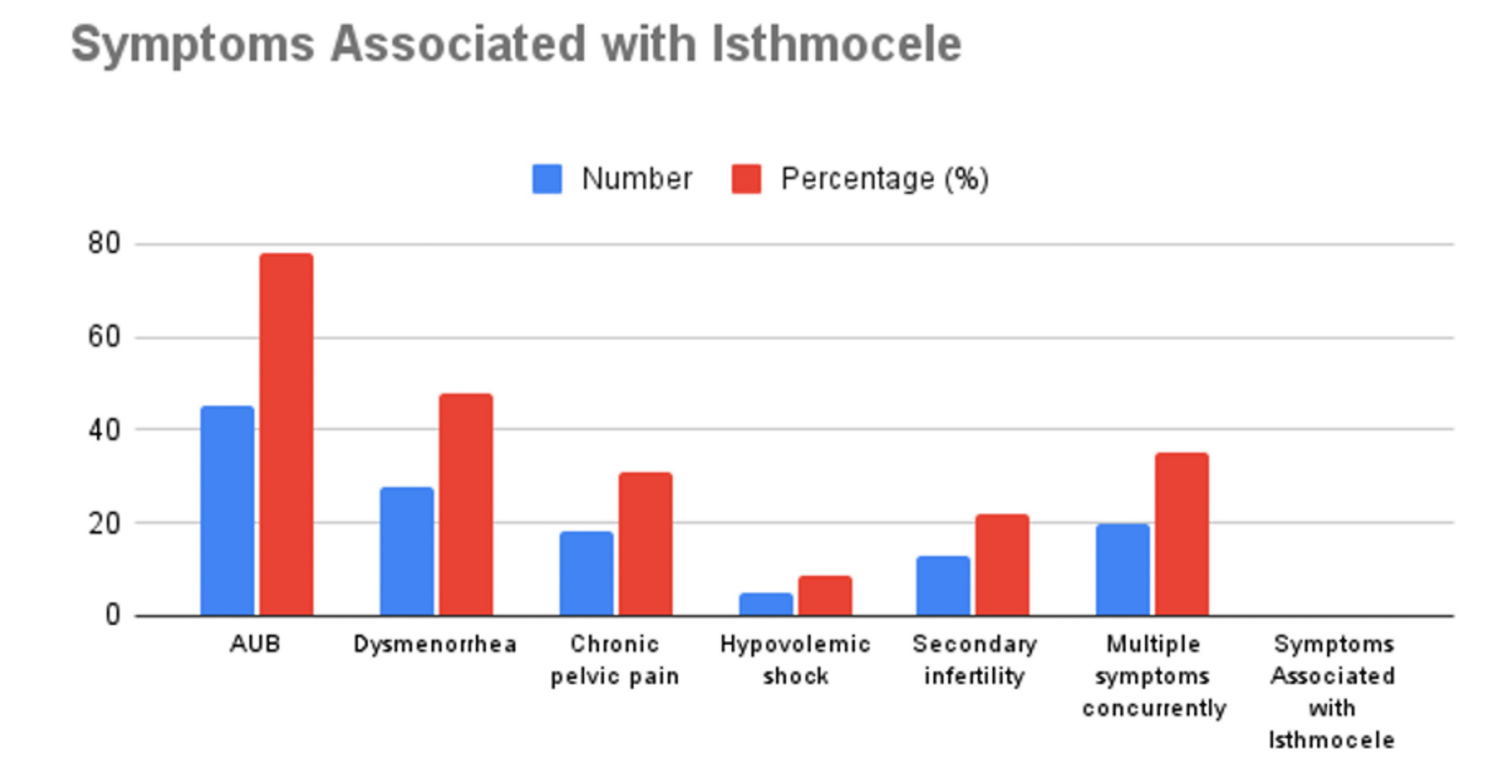
Symptoms prevalence associated with isthmocele in number and percentage Vaginal bleeding (78%) was the most common symptom, followed by dysmenorrhea (48%), chronic pelvic pain (31%), hypovolemic shock (8.6%), and secondary infertility (22%). Concurrent symptoms were present in 35% of cases, with 13.79% receiving both medical and surgical treatments.

The most frequently reported symptom among patients with uterine isthmocele was AUB, observed in 45 cases (78%), which showed a highly significant association (p = 0.00003). Dysmenorrhea was the second most common symptom, affecting 48% of patients; however, its association was not statistically significant (p = 0.793). Chronic pelvic pain was reported in 31% of cases and demonstrated a significant association with isthmocele (p = 0.004). Hypovolemic shock, although less common, was present in 8.6% of patients and was highly significant (p < 0.00001). Additionally, secondary infertility affected 22% of cases and was also highly significant (p = 0.00003). These findings highlight the diverse clinical presentation of isthmocele, emphasizing the importance of AUB and secondary infertility as key diagnostic indicators. Across both study phases, 58 cases of isthmocele were identified, with 13.79% of patients receiving a combination of medical and surgical interventions, while the majority were managed medically. The study findings emphasize AUB as the most significant clinical feature of isthmocele, with dysmenorrhea and chronic pelvic pain also commonly reported. Statistical analysis revealed a strong association between the development of isthmocele and the presence of three or more cesarean scars (p = 0.0001). The consistent incidence observed across the retrospective and prospective phases reflects the reliability of the diagnostic methods used in this study. These results underscore the importance of routine follow-up and targeted imaging for early detection and management of isthmocele, particularly in women with multiple cesarean scars. Figure 2 depicts the overall symptom associated with isthmocele in number (N) and in percentage (%).

## Discussion

This study represents the first comprehensive examination of isthmocele's prevalence and outcomes, particularly when associated with higher-grade cesarean sections. Isthmocele is an iatrogenic condition resulting from incomplete cesarean incision repair and reduced myometrial thickness, exacerbated by factors such as blood loss during surgery and additional hemostatic sutures [1,2]. While isthmocele often remains undiagnosed, 30-50% of cases present with symptoms like AUB, dyspareunia, chronic pelvic pain, dysmenorrhea, and secondary infertility [2,3]. Bleeding, frequently the primary symptom, often manifests as dark red or brown discharge post-menstruation, cesarean section spotting, or postcoital bleeding [1-3].

Our study at Rabia Balkhi Hospital, involving 9,207 women with previous cesarean scars, identified an isthmocele incidence rate of 0.628%. Among the 58 diagnosed cases, the most common symptoms were AUB (78%), dysmenorrhea (48%), and secondary infertility (22%). Similar to our findings, Baldini et al. (2024) reported an isthmocele prevalence of 0.6% in their cohort of 9,115 women, confirming the consistency of these rates across different populations [8]. Additionally, our study found that treating scarred areas without stitching yielded better healing outcomes, with ultrasonography proving to be an effective tool for evaluating cesarean scar repairs.

Comparatively, a meta-analysis by Murji et al. demonstrated a significant correlation between cesarean scar defects (CSD) and AUB, revealing a relative risk of 3.47 across six studies involving 1,385 participants [6]. The study further showed that the prevalence of AUB increased from 25% after one cesarean section to 76.4% in patients who underwent gynecologic imaging [6]. These findings corroborate our data, which highlighted AUB as the most prevalent symptom in 78% of cases [6]. Murji's meta-analysis also reported an average menstrual duration of 13.4 days, a figure similar to our observations in patients with more significant isthmocele defects [6].

A prospective study by Shabnam et al. (2023) revealed that 30% of women undergoing lower segment cesarean sections (LSCS) developed isthmocele [12], significantly higher than our incidence rate of 0.628%. Shabnam's study also identified key risk factors, including multiple cesarean deliveries, maternal BMI, and the characteristics of prior cesarean scars [12], which aligns with our findings that women with three cesarean scars experienced the highest incidence of isthmocele complications [12]. Furthermore, TVUS and SIS were found to be highly accurate in diagnosing isthmocele in Shabnam's study, supporting our use of ultrasound to diagnose isthmocele [12].

Recent research by Kulshrestha et al. (2020) underscored the importance of proper surgical techniques during cesarean sections to prevent isthmocele formation [13]. Their study found that using the far-far-near-near double-layer unlocked technique for uterine closure reduced the risk of isthmocele formation [13]. Our research contributes to the growing body of knowledge regarding isthmocele and its management. Treatment strategies may involve both medical and surgical interventions depending on the severity and depth of the defect [12,13]. Oral contraceptives and levonorgestrel intrauterine devices (LNG-IUDs) have been effective in managing symptoms such as AUB [13,14]. In more severe cases, surgical options ranging from hysteroscopic resection to complete hysterectomy may be required for women who no longer desire fertility [12-18]. Baldini et al. also noted that larger defects resulted in more significant blood accumulation and prolonged spotting, confirming our observation that defect size correlates with symptom severity [8].

This study emphasizes the importance of early diagnosis and appropriate surgical techniques to improve patient outcomes. Ultrasonography has proven effective for detecting CSD and is crucial for the early identification and management of isthmocele. Moving forward, our findings establish a foundation for future studies and practices in obstetrics and gynecology, with a focus on enhancing diagnostic methods and treatment pathways for this increasingly prevalent condition. Additionally, further exploration is required to understand genetic predispositions to isthmocele and the long-term outcomes of different management strategies. Advancements in imaging techniques and the establishment of standardized protocols for diagnosis and treatment are also crucial to improving patient care and outcomes. Expanding research across multiple centers will help broaden our understanding of the condition's prevalence and optimize its management.

Limitations and confounding factors

A key limitation of this study is the reliance on retrospective data, which may be prone to incomplete records and recall bias. Additionally, the study was conducted at a single hospital, potentially limiting the generalizability of the findings. Conducting future multicenter studies could validate these results and provide a broader perspective on the incidence and risk factors associated with isthmocele. Further limitations include the absence of systematic documentation on key variables such as the type of cesarean section performed, the presence of postoperative wound infections, and the specific timing of ultrasound evaluations. TVS was primarily used for imaging; however, no standardized grading criteria for isthmocele were applied, and details regarding the ultrasound examiners' training or experience were not specified. Additionally, variability in surgical techniques, individual healing responses, and follow-up intervals could have influenced the incidence and symptom outcomes observed. Overall, the lack of control over potential confounding factors highlights the need for future research employing standardized diagnostic criteria, prospective data collection, and detailed evaluation of the effects of various surgical techniques on isthmocele development.

## Conclusions

This four-year observational study at Rabia Balkhi Hospital provides valuable insights into the prevalence and clinical features of uterine isthmocele among women with previous cesarean sections. The combined retrospective and prospective approaches allowed for a comprehensive analysis of patient records and real-time evaluations, highlighting the incidence, symptomatology, and diagnostic challenges associated with isthmocele. The overall incidence of isthmocele was found to be 0.62% among women with prior cesarean sections, with AUB being the most commonly reported symptom. The study also identified a higher prevalence of isthmocele in patients with multiple cesarean scars, emphasizing the need for careful monitoring and follow-up in this population. Future research should focus on developing standardized diagnostic protocols, exploring the long-term impact of isthmocele on reproductive health, and addressing the challenges of implementing artificial intelligence-driven diagnostic tools in low-resource settings. Interdisciplinary collaboration between clinicians, radiologists, and artificial intelligence experts will be crucial in advancing the understanding and management of isthmocele, ultimately enhancing the quality of care for women with previous cesarean sections.
